# A Case of *CCDC6-RET* Fusion Mutation in Adult Acute Lymphoblastic Leukemia (ALL), a Known Activating Mutation Reported in ALL

**DOI:** 10.3389/fonc.2019.01303

**Published:** 2019-12-03

**Authors:** Mateo Mejia Saldarriaga, Amir Steinberg, Eric A. Severson, Adam Binder

**Affiliations:** ^1^Albert Einstein College of Medicine/Jacobi Medical Center, The Bronx, NY, United States; ^2^Department of Oncology, Mount Sinai Hospital, New York, NY, United States; ^3^Foundation Medicine, Morrisville, NC, United States; ^4^Department of Oncology, Sidney Kimmel Cancer Center, Thomas Jefferson University, Philadelphia, PA, United States

**Keywords:** acute lymphoblastic leukemia, RET, CCDC6-RET, novel mutation, FoundationOneHeme

## Abstract

We report the case of a patient with B-Cell Acute Lymphoblastic Leukemia (ALL) who was found to harbor a gene fusion involving the *CCDC6* and *RET* genes. Although the *RET* mutations have been identified before in other malignancies, and it is thought to represent a driver mutation in these neoplasms, it has yet to be described in ALL. The identification of known fusion genes conferring activating tyrosine kinase activity in neoplasms can suggest potential therapeutic role of tyrosine kinase inhibitors (TKI), an approach that has been exploited in several other fusion genes.

## Introduction

Sincethe discovery of a recurrent translocation in patients with chronic myeloid leukemia, t(9:22)(q34;q11), later identified as the *BCR-ABL* fusion gene, the role of gene fusions involving tyrosine kinases have been recognized as important oncogenic drivers.In ALL, the t(9:22) translocation is the most frequent cytogenetic abnormality. *BCR-ABL*+ ALL patients comprise a distinct clinical subgroup (referred as Ph+ ALL), with a more severe clinical presentation, historically poorer prognosis, and more prevalent in adults than children (1). Therapeutic targeting of the *BCR-ABL* fusion gene with TKI has improved treatment response and overall survival for patients with Ph+ ALL. We describe here the case of a 55 year-old female who presented with pancytopenia, lymphadenopathy, and was found to have ALL with a *CCDC6-RET* fusion. Understanding the role of the different genomic alterations present in ALL is of crucial importance, as they have prognostic implications and are evolving therapeutic targets. The first targeted therapy in ALL was against the *BCR-ABL* fusion protein, which improved overall response and survival in “fit” patients, but also allowed a promising alternative treatment strategy for elderly or frail patients (2, 3). More recently, a subgroup of patients were noticed to share the same gene expression profile as Ph-positive, but lack the *BCR-ABL* translocation. These patients have several different genomic alterations, and are possibly targeted using different TKI's (4). *RET* gene fusions and point mutations are known driver mutation in several neoplasms, including papillary thyroid cancer (PTC), non-small cell lung cancer (NSCLC), acute myeloid leukemia, and sporadic medullary thyroid cancer. *RET* gene fusions are the main oncogenic driver in patients with multiple endocrine type 2 (5). *RET* gene fusions have not previously been described in patients with ALL (6, 7).

## Case Report

A 55 year-old female patient with past medical history of type 2 diabetes, hypothyroidism and opioid dependence presented to the ED with 2 weeks of right flank pain radiating to her right groin. Her initial vital signs were stable, and she was afebrile. Her physical exam was remarkable for bruising in her lower extremities and a hemorrhagic bulla under her tongue. Palpation of her back revealed right costovertebral angle tenderness. Lymph node exam demonstrated a few bilateral shotty inguinal lymph nodes.

The patient's initial blood work was remarkable for pancytopenia (white blood cell count 2.1 ×10^6^/uL, Hb 112 g/L, platelets 0.03 ×10^9^/uL), with normal renal function and elevated Alkaline Phosphatase, AST and ALT (265, 481, and 39 U/L, respectively). Lactate dehydrogenase (LDH) was severely elevated 17,216 U/L, with normal coagulation time, fibrinogen and uric acid. A bone marrow flow cytometry revealed a population of blasts that represented 77% of the sample. The blast were positive for CD9, CD19, CD10 (variable), CD20, cCD22, CD34, CD45, cCD79a, HLA-DR, and cTdT. The blasts were negative for: CD2, cCD3, CD4, CD5, CD7, CD8, CD1a, CD11b, CD11c, CD13, CD14, CD25, CD33, CD56, CD64, CD91, CD117, CD123, CD163, cIgM, cMPO, Kappa, and Lambda. These findings were compatible with B-lymphoblastic leukemia. BCR/ABL was negative by PCR, and cytogenetics were abnormal with 71,74,XXXX,XXX,XX,-3,+4,+5,+6,+6,+6,-7,-7,+8,-9,-9,+10,-10,+11,-12,-12,-13,-14,-14,+15,+15,-16,-16,-17,-18,-18,+19,+20,+20,-20,+21,+22,+22,+22,del(22)(q11.2),x2[cp15]/46,XX [5].

Patient completed induction chemotherapy with R-HyperCVAD, with complete remission as determined by bone marrow biopsy at the end of the first cycle. Course was complicated by neutropenic fever after the first cycle. FoundatioOne®Heme was sent as part of her diagnostic work up and revealed the following genomic alterations: *CCDC6-RET* gene fusion, *FLT3* D835H and D835V, *CDKN2A/B* loss, *DNMT3A* truncation in intron 14, *KMT2C* S793^*^ (encoding MLL3) and *TP53* K132R. The patient finished induction with negative minimal residual disease (MRD) by flow cytometry and resolution of cytogenetic abnormalities observed at diagnosis, and underwent myeloablative allogenic bone marrow transplant from peripheral blood donor, with cyclophosphamide and total body irradiation conditioning regimen.

Her initial post-transplant course was uncomplicated initially; however, the patient developed acute graft vs. host disease involving the skin, with a good response to steroids. Ruxolitinib was introduced later as steroid sparing agent. To date, 500 days since transplant, she continues to be in remission, with no MRD in last assessment 1 year post-transplant. Patient authorized and consented use of her information and publication of the case.

## Discussion

The detection of new potential driver mutations in different neoplasms has been boosted by the introduction of several comprehensive genomic profiling assays available for commercial use. These assays both identify known driver mutations as well as novel genomic alterations.

Activating mutations involving tyrosine kinase receptors and other molecules involved in intracellular signal amplifications/transduction are known to be important drivers of oncogenesis. Moreover, the presence of the same mutation across different neoplasm, often represents an important pharmacological target. *CCDC6-RET* and other common fusion partners leads to homodimerization through coiled-coil interactions in the fusion partner protein, preserving the tyrosine kinase activity in the RET intracellular domain and causing ligand independent activation (9). RET autophosphorylation lead to activation of several intracellular transduction cascades involved in cellular proliferation, including MAPK, AKT, PKC, JAK-STAT, PKA and PI3K (5).

**Figure 1:**
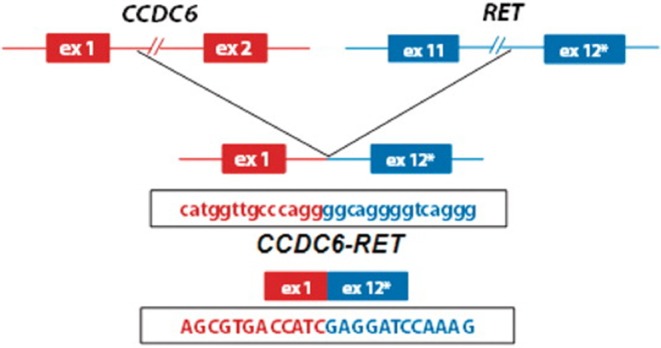
Schematic representation of the CCDC6-RET fusion gene identified in patients with papilary thyroid cancer (8) (used with permission, copyright Nucleic Acids Research, Oxford University Press).

*RET* gene fusions were first identified in patients with PTC, followed by a subset of patients with NSCLC (10). This mutation has been targeted recently with TKIs, offering a new line of treatment for these patients (Figure 1) (11). *CCDC6-RET* translocations are the most common type of *RET* fusion present in PTC (12). These mutations are considered to have a considerable role in oncogenesis as they are usually mutually exclusive with other alterations such as *BRAF* and *RAS* in PTC (13), a hypothesis that is supported by clinical response to TKIs (5). More recently, *RET* gene fusions, including *CCDC6* have been identified in NSCLC (10), resulting in clinical trials for TKIs in this subgroup (11, 14).

Several multi-kinase inhibitors with *RET* inhibition are available and have been approved for several different indications. Vandetanib (14), Lenvatinib (15), Alectinib (16), and Cabozatinib (11) have been used in trials of NSCLC with rearranged *RET*. Ponatinib (9), Sunitinib (7), Sorafenib (17), and Axitinib (5) are molecules that inhibit multiple tyrosine kinase proteins, but also exhibit multitarget *RET* inhibition, and have been used in different clinical settings. Newer *RET* specific molecules (BLU-667, LOXO-292) are currently being developed and evaluated in different clinical scenarios (5).

Despite the evidence for the central role of *RET* as a driver mutation, different agents inhibiting *RET* in NSCLC and PTC have showed different degrees of overall response rate, ranging from 0 to 50% (5). Several hypotheses for this heterogeneous effect exist, including significant differences in potency (as reflected by IC_50_), different types of interaction with the ATP-binding pocket of *RET*, and differences in non-*RET* tyrosine kinase inhibition. The latter mechanism is especially of interest given the preclinical evidence of resistance to *RET* inhibition through *EGFR*-pathway activation in NSCLC, suggesting a potential rational for multiple tyrosine kinase inhibition (18).

Additionally, the *RET* fusion partner gene might impact the expression of *RET* despite not involving the tyrosine kinase domain. Reports of different degrees of *RET* expression varies, with a higher degree of *RET* expression in *KIF5B-RET* when compared to *CCDC6-RET*. Additionally, *KIF5B-RET* has been associated with multikinase activation independent of the *RET*-tyrosine kinase (19). Evidence of heterogeneous clinical activity of Vandetanib across different *RET* fusion partners has been observed in clinically in phase 2 trials in NSCLC (14, 20).

The previously described additional mechanism in *RET* mutations might explain the significant gap observed in patients with actionable NSCLC such as *EGFR, ALK, ROS1*, and *RET*, where the response rate and progression free survival is roughly half for *RET* inhibitors (5).

As described in NSCLC, dependence on other pathways could also potentially lead to resistance of *RET* inhibition. In this case, other associated mutations such as *FLT3, DNMT3A, MLL*, and *TP53* act as oncogenes or tumor suppressor genes could further contribute to resistance, as they involve other tyrosine kinase proteins (*FLT3*), important epigenetic (*DNTM3A, MLL3*) and DNA damage-response elements (*TP53*). Off note, *FLT3* D835 mutations have been described before in pediatric ALL (21), and is one of the most frequent mutations in acute myeloid leukemia (AML). *FLT3* is a well-recognized target in AML and TKIs such as midostaurin and gilteritinib are approved to target this mutation (22). *DNMT3A* mutations have been described in 5% of 57 patient with ALL, predominantly of T-cell origin, and are thought to lead to worse outcomes, although there is no information in large cohorts (23). Although these mutations are known to be present in ALL, the interactions with *CCDC6-RET* is unknown and their overall prognostic implication is unclear.

## Conclusion

This is the first report of *CCDC6-RET* rearrangement in ALL to date. The increased use of genome wide comprehensive assays is an important tool to discover novel genomic alterations and genomic alterations common in certain malignancies in unexpected contexts. These findings may be suitable for targeted therapies. The isolation of *CCDC6-RET* fusion raise the possibility of directed *RET* inhibition with selective or multitarget TKI in this disease. Drugs such as ponatinib and sorafenib are already used for patients with underlying hematologic malignancies, with a well-studied safety profile; particularly ponatinib in Ph+ ALL. Fortunately, our patient continues to be in remission post-transplant, but this could be a treatment option in the future should she relapse. Comprehensive genomic profiling including interrogation of *RET* rearrangement and point mutations should be conducted in ALL to identify the prevalence and clinical implications of this new finding. Further characterization of *RET* activation and resistance mechanism are required to assess for better strategies to improve clinical outcomes in this subset of malignancies.

## Ethics Statement

Analysis and data gathering was purely retrospective and with consent by the patient.

## Disclosure

ES is employed by Foundation Medicine, Inc. Part of this case was presented as an online abstract at American Society of Hematology annual meeting 2018 at San Diego, California.

## Author Contributions

AB and AS participated directly in the clinical care of the patient. All authors participated actively in textual elaboration.

### Conflict of Interest

ES is an employee at Fundation Medicine. The remaining authors declare that the research was conducted in the absence of any commercial or financial relationships that could be construed as a potential conflict of interest.
